# Pertussis seasonal variation in Northern Vietnam: the evidence from a tertiary hospital

**DOI:** 10.1186/s12889-024-17705-9

**Published:** 2024-01-24

**Authors:** Nhung TH Pham, Quyen TT Bui, Dien M Tran, Mattias Larsson, Mai P Pham, Linus Olson

**Affiliations:** 1 Infection Prevention and Control Department, Vietnam National Children’s Hospital, Hanoi, Vietnam; 2 Field Epidemiology Training Program - Ministry of Health, Hanoi, Vietnam; 3 Faculty of Fundamental Sciences, Hanoi University of Public Health, No. 1A Duc Thang Ward, North Tu Liem, Ha Noi, Vietnam; 4 Vietnam National Children’s Hospital, Hanoi, Vietnam; 5 Pediatric Department, School of Medicine and Pharmacy, Vietnam National University, Hanoi, Vietnam; 6 Department of Public Health Sciences, Karolinska Institutet, Stockholm, Sweden; 7 Training and Research Academic Collaboration, Sweden-Vietnam, Stockholm, Sweden; 8 School of Preventive Medicine and Public Health, Hanoi Medical University, Hanoi, Vietnam; 9 Department of Women’s and Children’s Health, Karolinska Institutet, Stockholm, Sweden

**Keywords:** Pertussis, Seasonal variation, Children, Outbreak cycles, Severity

## Abstract

**Background:**

Pertussis is a highly contagious and dangerous respiratory disease that threatens children’s health in many countries, including Vietnam, despite vaccine coverage. From 2015 to 2018, Vietnam experienced an increasing number of pertussis patients. Therefore, this study aimed to investigate the trend and examine the seasonal variations of pertussis in North Vietnam.

**Methods:**

Data were collected from medical records of all under-5-year-old inpatients admitted to the National Children’s Hospital in Hanoi, Vietnam (VNCH) 2015–2018. A descriptive analysis was performed to describe the distribution of incident cases by year and season. Linear multivariable regression was conducted to investigate the association between the incidence of cases and seasonality adjusted by age and vaccination status.

**Results:**

We identified 1063 laboratory-confirmed patients during 2015–2018, including 247 (23.2%) severe patients. The number of pertussis patients admitted to VNCH per 1000 hospitalizations was 3.2 in 2015, compared to 1.9, 3.1, and 2.1 in 2016, 2017, and 2018, respectively. Outbreaks occurred biennially; however, there was no significant difference in the number of severe patients over this period. Most cases occurred in the hot season (509 patients, or nearly half of the study population). With the adjustment of the vaccination rate and average age, the risk of pertussis-associated hospitalization in the mild season and the hot season was 21% (95% CI [0.12; 0.3]) and 15% (95% CI [0.05; 0.25]) higher than that in the warm season, respectively. The rate of hospitalizations was high in the mild season (28.9%) and the warm season (30.8%), nearly twice as much as that in the hot season; nevertheless, the death rate was only striking high in the mild season, about 5–6 times as much as those in the other seasons.

**Conclusion:**

The pertussis incidence in Northern Vietnam varied between seasons, peaking in the hot season (April-July). However, severe patients and deaths increased in the mild season (December-March). Interventions, for example, communication activities on pertussis and vaccination, are of immense importance in lowering the prevalence of pertussis. In addition, early diagnoses and early warnings performed by health professionals should be encouraged.

## Introduction

Pertussis is a highly contagious respiratory but vaccine-preventable disease. Vaccination against diphtheria, pertussis (whooping cough), and tetanus (DPT, whole cell vaccination) among children has globally decreased the incidence, severity, and mortality rate of pertussis. Before introducing the pertussis vaccine, the World Health Organization (WHO) estimated more than one million patients of pertussis in seven years, with an annual incidence of 150 patients per 100,000 people worldwide. This figure has declined by more than 80% (1 to 8 per 100,000 people) compared with the pre-vaccine period [1]. However, pertussis remains a major health problem, with 195,000 pediatric deaths and epidemics persisting globally [2]. In 2012, the United States reported a record number of pertussis patients in recent years (48,277). In 2016, Denmark experienced an epidemic with 2096 laboratory-confirmed patients of pertussis, most of whom were children under 12 months old [3]. In early 2017, using electronic Communicable Disease Surveillance (e-CDS) software, Vietnam observed an increase in pertussis across alleges (563 patients), mainly among children under three months old in North Vietnam (435 patients), with a caseload of 1.3–2 times higher than the average [4].

Seasonality is a common feature of infectious diseases: Influenza outbreaks peak during winter in temperate Europe and North America, as viruses are more stable under cold and dry conditions. Chickenpox [5, 6] epidemics peak each spring in Europe and at various times in South America [6, 7]. Information about the seasonality of infectious diseases, especially person-to-person-transmitted diseases, is a public health concern. A better understanding of seasonality will contribute to improving the accuracy of public health surveillance, supporting the early detection of outbreaks, and enhancing the outbreak response measures of forecasting systems. Additionally, it could inform the development of cost-effective prevention and treatment measures [8, 9]. Similar to the epidemics of other infectious diseases, pertussis reoccurs at 2–3 years intervals and peaks every 3–4 years. However, the seasonality of pertussis outbreaks remains inconsistent [10, 11]. North Vietnam had a high pertussis incidence of 324 patients in 2015 compared to 435 patients in 2017, according to the Vietnam Health Statistic Yearbook 2015–2018 [12]. The Vietnam National Children’s Hospital (VNCH), a leading medical facility in northern Vietnam, reported high numbers of pertussis patients in these two years. Our current study aims to describe the distribution and trends in pertussis epidemiological and clinical characteristics by season over four years from 2015 to 2018. Its results are expected to provide evidence for policies on prevention, diagnosis, and treatment methods.

## Methodology

### Study design and setting

The cross-sectional study was conducted at Vietnam National Children’s Hospital (VNCH) from January 2020 to June 2022.

### Study population

A list was made of all pertussis in-patients under five years old with a discharge diagnosis confirmed by a positive polymerase chain reaction (PCR) or immunoglobulin M (IgM) test from 2015 to 2018. A positive PCR test involves detecting genetic material from *B. pertussis* on a nasopharyngeal swab or in aspirate from suspected cases. A positive IgM test means specific IgM antibodies against *Bordetella pertussis* are detected and quantitatively determined in a blood sample.

### Sampling and data collection

All records of pertussis inpatients from 2015 to 2018 were collected. Data were extracted from the hospital patient management software using a structured questionnaire. This study categorized all inpatients who required medical oxygen (given through mask for longer than 24 h), a ventilator (for longer than 24 h), dialysis, or extracorporeal membrane oxygenation (ECMO) as severe cases.

Since 2014, the VNCH has implemented two surveillance systems to collect and report data on infectious diseases. Nurses in each clinical department documented all hospitalizations due to pertussis and other diseases in the admission and discharge register. These data are reported monthly and then used by the General Department to compile a written report submitted to the Department of Medical Service Administration, Ministry of Health (MOH). In addition, the Infectious Disease Department nurses and the Intensive Care Unit fill in the patient information in electronic questionnaires, which are directly sent to the General Department of Preventive Medicine under Circular 54 of the Ministry of Health (National Notifiable Disease Surveillance System (NNDSS). From 2015 to 2018, the hospital provided medical services for approximately six to twelve thousand admissions each month. The study analyzed data for long-term trends (years) to examine the general seasonal pattern of pertussis disease in the VNCH and North Vietnam. This paper provides a detailed description of the data collection process and the definitions of cases and severe cases in the study [13].

### Study variables and measurement tool

#### Sociodemographic characteristics included age and vaccination

A patient’s age was calculated from their date of birth to the onset date of symptoms, and they were classified into age groups according to the DTP vaccination schedule for children. The vaccination status was defined as vaccinated, no vaccination, and late vaccination. They were referred as vaccinated if the child received the last dose of vaccine at least one month before the onset of symptoms and followed the recommended vaccination schedule. If the child had less than one month between the date of vaccination and onset, their status was defined as “no” vaccination. If a child did not receive the vaccine doses before or at the age of vaccination and a one-month gap existed between the time of vaccination and the onset of pertussis symptoms, it was considered a “late” vaccination.

#### Onset symptoms

Paroxysms of coughing, OR inspiratory whoop, OR post-tussive vomiting, OR apnea (with or without cyanosis).

#### Seasonal variation

Based on specific characteristics such as temperatureand rainfall, which are considered factors that can influence the trend of pertussis disease [14, 15], the seasons in Northern Vietnam in this study have been split into three equally long (120 ± 2 days) seasons: (1) December to March - mild weather with occasional cold spells (mild season): with average daytime temperatures is 21.8^0^C, 3.2 sunshine hours per day, and 4.7 rain days per month (a rain day is a day on which at least 0.1 mm precipititation); (2) April to July - hot and rainy (hot season): with average daytime temperatures is 30.9^0^C, 5.3 sunshine hours per day, and 10.9 rain days per month; and (3) August to November - warm with occasional typhoons (warm season): with average daytime temperatures is 29.0^0^C, 5.1 sunshine hours per day, and 8.7 rain days per month [15].

### Statistical approach

Data were analyzed using STATA 15.0 (StataCorp LLC, 4905 Lakeway Drive, College Station, Texas 77,845 − 4512, USA) software. The onset of the first symptoms was used to classify the cases by month, season, year, and region.

The date of disease occurrence was defined as symptom onset; if the date of commencement was missing, the date of disease occurrence was that of the pertussis test. We compared the sociodemographic and clinical characteristics, the duration from onset to test, the length of hospitalization, and in-hospital outcomes of patients over the four years and seasons with Kruskal–Wallis tests for continuous variables and chi-square or Fisher tests for categorical variables. Additionally, the significance cutoff was a *p*-value of less than 0.05. Time-series analysis was used to present the monthly incidence over four years. Univariate linear regression models were performed to determine the associations of season factors with the percentage of pertussis among hospitalizations by month. Meanwhile, the multivariate linear regression model was also applied to control for mean age and vaccination percentage among hospitalized pertussis cases.

### Ethical consideration

Ethical approval was received from the ethical review board of Vietnam National Children’s Hospital with a waiver of patient consent. Patient records and abstracted information were anonymized and deidentified before analysis, and the study followed the latest guidelines stated in the Declaration of Helsinki.

## Results

**Fig. 1:**
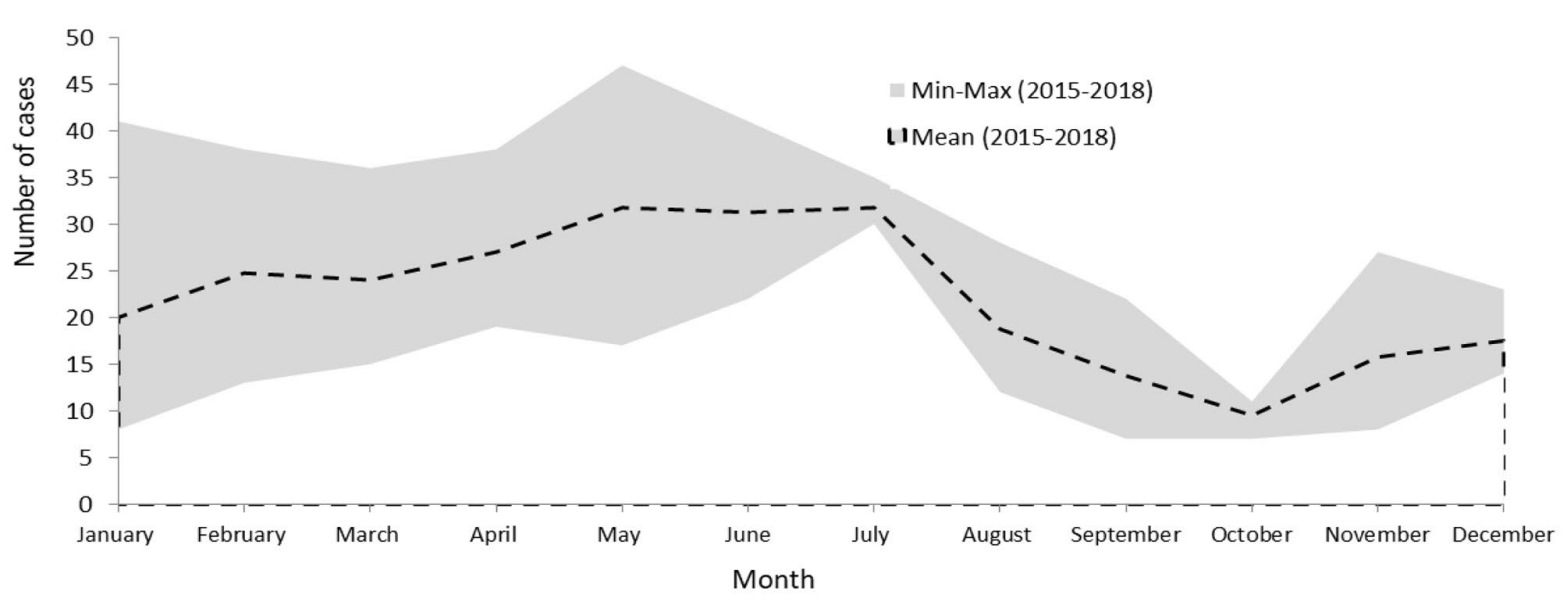
Monthly distribution of hospitalized pertussis patients in VNCH, 2015–2018 (*N* = 1063)

From 2015 to 2018, VNCH treated 1063 inpatients diagnosed with pertussis. Figure 1 shows the distribution of hospitalized pertussis patients in 2015–2018 by month, with the highest number of patients reported in May, June, and July (hot season). October is the month with the lowest number of patients.

**Fig. 2:**
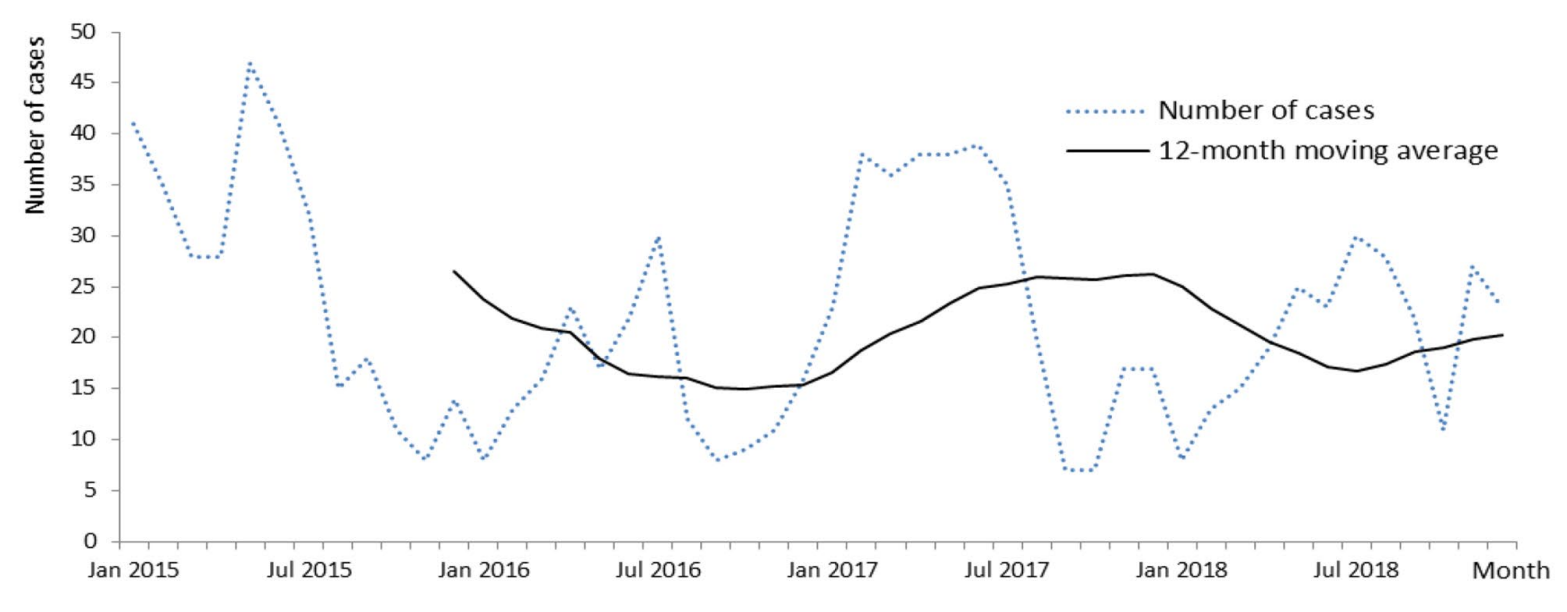
Trend and number of hospitalized pertussis patients in VNCH, 2015–2018 (*N* = 1063)

Figure 2 shows the trend of admitted pertussis patients in the VNCH, 2015–2018. The number of reported patients decreased from 2015 to 2016, followed by an increase in 2017.

**Fig. 3:**
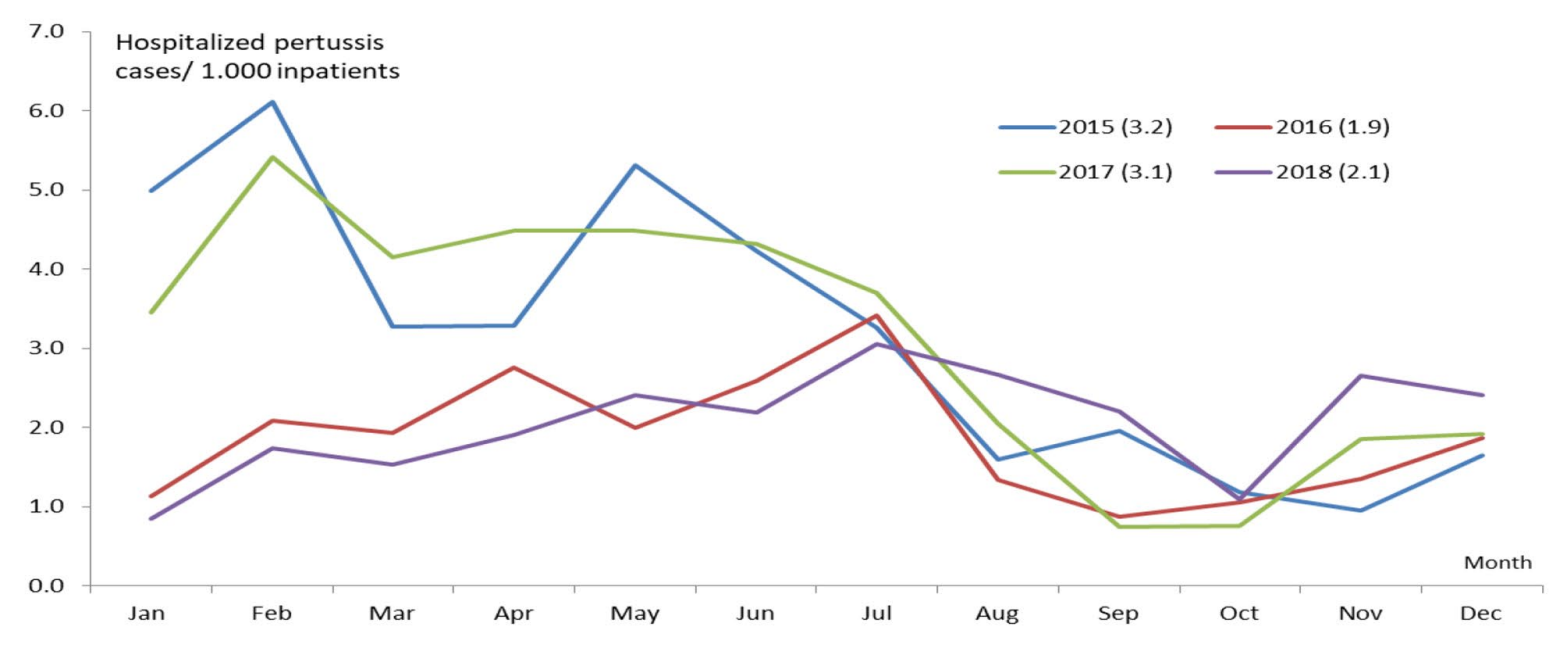
Incidence of pertussis per 1,000 inpatients in VNCH by year (2015–2018)

Figure 3 shows the incidence of pertussis patients per 1,000 inpatients seeking care in VNCH annually. The annual incidence ranged from 1.9 patients in 2016 to 3.2 in 2015. There were high incidences in the first halves of 2015 and 2017 (with a peak in February); the number of cases decreased from May or June onward. The situation was different for 2016 and 2018: the number of pertussis cases per 1,000 inpatients slightly increased from the beginning of the year to July (with a peak in July) before experiencing a decrease.

Among 1063 pertussis patients, males accounted for 50.1% of the admissions (Table 1). The median age of hospitalized pertussis patients was 2.2 years (IQR: 1.1–4.1 years); there was a significant difference in the median age of pertussis patients across years (*p* < 0.001), the highest median age of the patient was 2.5 in 2018 and the lowest was 2.0 in. About 44% of patients were younger than two months, and those under one year old accounted for 88% of all hospitalized pertussis patients. Children’s ages reported in 2018 were slightly younger than those reported in 2015–2017. Nearly half of the admissions (*n* = 509; 47.9%) occurred in the hot season (April through July), followed by the mild season (*n* = 398; 37.4%) and the warm season (*n* = 156; 14.7%).

**Table 1:** Characteristics of hospitalized pertussis patients in VNCH by year (2015–2018)

Characteristic	Total (*N* = 1063)	2015 (*n* = 318)	2016 (*n* = 185)	2017 (*n* = 316)	2018 (*n* = 244)	*p* value
**Age month: median (IQR)**	2.2 (1.1; 4.1)	2.2 (1.2; 3.7)	2.4 (1.4; 4.4)	2.5 (1.3; 6.4)	2.0 (1.0; 4.0)	< 0.001^*ƒ*^
**Age group**						
< 2 m	468(44.0)	146(45.9)	84(45.4)	132(41.8)	106(43.4)	-
2 m–<3 m	207(19.5)	65(20.4)	35(18.9)	49(15.5)	58(23.8)	
3 m–<4 m	117(11.0)	44(13.8)	19(10.3)	37(11.7)	17(7.0)	
4–<5 m	51(4.8)	15(4.7)	8(4.3)	16(5.1)	12(4.9)	
5–<12 m	92(8.7)	28(8.8)	24(13.0)	27(8.5)	13(5.3)	
1–<5 y	128(12.0)	20(6.3)	15(8.1)	55(17.4)	38(15.6)	
**Sex (Male)**	533(50.1)	169(53.1)	94(50.8)	143(45.3)	127(52.0)	0.207^*†*^
**Seasons (total number, % of cases that year)**						
Mild (Dec-Mar)	398(37.4)	128(40.3)	69(37.3)	133(42.1)	68(27.9)	< 0.001^*†*^
Hot (Apr–Jul)	509(47.9)	153(48.1)	88(47.6)	152(48.1)	116(47.5)	
Warm (Aug–Nov)	156(14.7)	37(11.6)	28(15.1)	31(9.8)	60(24.6)	
**At least one vaccination dose**	172(16.2)	43(13.5)	28(15.1)	87(27.5)	14(5.7)	< 0.001^*†*^
**Vaccination groups (total number, % of cases that year)**						
0 doses	891(83.8)	275(86.5)	157(84.9)	229(72.5)	230(94.3)	-
1 dose	83(7.8)	31(9.7)	15(8.1)	27(8.5)	10(4.1)	
2 doses	18(1.7)	4(1.3)	4(2.2)	9(2.8)	1(0.4)	
3 doses	23(2.2)	3(0.9)	4(2.2)	15(4.7)	1(0.4)	
> 3 doses	48(4.5)	5(1.6)	5(2.7)	36(11.4)	2(0.8)	
**Prolonged cough**	879(96.8)	315(99.1)	184(99.5)	294(93.0)	86(96.6)	< 0.001^*‡*^
**Severe cough**	652(71.8)	254(79.9)	142(76.8)	191(60.4)	65(73.0)	< 0.001^*†*^
**Severity**	247(23.2)	60(18.9)	43(23.2)	74(23.4)	70(28.7)	0.058^*†*^
**Fatal**	20(1.9)	4(1.3)	6(3.2)	7(2.2)	3(1.2)	0.353
**Duration time from onset to test; Median (IQR)**	10(7–15)	11(7–18)	11(6–15)	10(6–15)	10(10–10)	0.016^*ƒ*^
**Length of hospitalization; Median (IQR)**	9(6–14)	8(5–12.5)	10(6.0–17)	10(6–16)	9(6–14)	< 0.001^*ƒ*^

^ƒ^Kruskal–Wallis test. ^†^Chi-square. ^‡^Exact test. IQR: interquartile range; m: months; y: year

Regarding the vaccination status of pertussis patients (Table 1), 172 patients (16.2%) received at least one vaccine dose, and there was a significant difference between years (the vaccination rate was found to be highest in 2017 and lowest in 2018- *p* < 0.001). Patients who were given one dose comprised approximately 7.8%, compared to 1.7% with two doses and 6.7% with three or more doses.

Most patients experienced prolonged coughing (96.8%), approximately three-fourths of whom suffered critical coughing. Among the 1063 children hospitalized for pertussis, 247 had severe symptoms (23.2%). The rate of severe patients was highest in 2018 (70 patients, accounting for 28.7% of all pertussis patients identified in this same year) and lowest in 2015 (60 patients, 18.9%); *p* < 0.001.

The overall case fatality rate was 1.9% (*n* = 20), with the highest and lowest rates of 3.2% and 1.2% in 2016 and 2018, respectively. However, those case fatality rates were not significantly different by year (*p* > 0.05). As shown in Table 1, the median length of stay was nine days (IQR: 6–14 days), and the median duration from the onset of symptoms to the pertussis test was ten days (IQR: 7–15 days). There was a significant difference between the years in term of length of stay in the hospital (*p* < 0.001).

Table 2 showed the characteristics of hospitalized pertussis patients by season. There were no significant differences across seasons regarding sex, region, duration from onset to test, or length of hospitalization (*p* > 0.05). Mechanical ventilation was significantly more common (*p* = 0.001) in the warm and mild seasons, 10.3% and 9.8%, respectively, compared to only 3.9% (*n* = 20) in the hot season. Overall, this trend was also observed in severe cases. The highest rate of severe cases was 30.8% among patients hospitalized in the warm season, followed by 28.9% and 16.5% in the mild and hot seasons, respectively. Notably, three-quarters of the fatalities (15 cases) were hospitalized in the mild season (Table 2). The case fatality rate in the mild season was 3.8%, followed by 0.8% in the hot season and 0.6% in the warm season.

**Table 2:** Characteristics of hospitalized pertussis patients in VNCH by season (2015–2018)

Characteristic/symptoms	Total (*N* = 1063)	Dec–Mar (Mild season) (*n* = 398)	Apr–Jul (Hot season) (*n* = 509)	Aug–Nov (Warm season) (*n* = 156)	*p* value
**Age (median, IQR)**	2.2(1.1–4.1)	2.0 (1.2–3.5)	2.3 (1.1–4.8)	2.0(1.0–9.3)	0.010^*ƒ*^
**Age group (total number, % of cases that year)**					
< 2 m	468(44.0)	193(48.5)	208(40.9)	67(42.9)	< 0.001^*†*^
2 m–<3 m	207(19.5)	77(19.3)	101(19.8)	29(18.6)	
3 m–<4 m	117(11.0)	55(13.8)	51(10.0)	11(7.1)	
4–<5 m	51(4.8)	16(4.0)	32(6.3)	3(1.9)	
5 m–<12 m	92(8.7)	28(7.0)	54(10.6)	10(6.4)	
1–<5 y	128(12.0)	29(7.3)	63(12.4)	36(23.1)	
**Sex (Male)**	533(50.1)	205(51.5)	251(49.3)	77(49.4)	0.788^*†*^
**Vaccination Group (total number, % of cases that year)**					
0 doses	891(83.8)	336(84.4)	419(82.3)	136(87.2)	0.008^*‡*^
1 dose	83(7.8)	41(10.3)	39(7.7)	3(1.9)	
2 doses	18(1.7)	4(1.0)	11(2.2)	3(1.9)	
3 doses	23(2.2)	4(1.0)	16(3.1)	3(1.9)	
> 3 doses	48(4.5)	13(3.3)	24(4.7)	11(7.1)	
**Prolonged cough**	879(96.8)	359(95.7)	428(97.9)	92(95.8)	0.173^*†*^
**Severe cough**	652(71.8)	286(76.3)	302(69.1)	64(66.7)	0.039^*†*^
**Mechanical ventilation**	75(7.1)	39(9.8)	20(3.9)	16(10.3)	0.001^*†*^
**Severity**	247(23.2)	115(28.9)	84(16.5)	48(30.8)	< 0.001^*†*^
**Fatal**	20(1.9)	15(3.8)	4(0.8)	1(0.6)	0.004^*‡*^
**Duration time from onset to test; (median, IQR)**	10.0(7–15)	10.0 (7.0 − 15.0)	10.0(7.0 − 14.0)	10.0(10.0–14.0)	0.214^*ƒ*^
**Length of hospitalization; (median, IQR)**	9.0(6–14)	10.0(6.0–14.0)	9.0(6.0–14.0)	9.0(6.0–16.0)	0.807^*ƒ*^

^ƒ^Kruskal–Wallis test. ^†^Chi-square. ^‡^Exact test; IQR: interquartile range; m: months; y: year

Table 3 showed the change in the pertussis incidence by season. When the proportion of vaccination and the average age of children were controlled, the rates of pertussis hospitalization in the mild season (Dec-Mar) and the hot season were 15% (95% CI [0.05; 0.24]) and 21% (95% CI [0.12; 0.30]) higher than those in the warm season, respectively.

**Table 3:** Multivariable linear regression of the seasonal variation in pertussis incidence

Characteristic	Univariate analysis		Multivariate analysis	
	Coeff.	95% CI	Coeff.	95%CI
**Seasons**				
Warm season	Ref.		Ref.	
Mild season	0.11	0.03; 0.20	0.15	0.05; 0.25
Hot season	0.18	0.09; 0.27	0.21	0.12; 0.30
**% of vaccination**	0.00	-0.003; 0.003	− 0.00	-0.004; 0.003
**Average age**	-0.001	-0.01; 0.01	0.01	-0.01; 0.02

## Discussion

The National Children’s Hospital in Hanoi, Vietnam is the leading pediatric hospital in the northern part of Vietnam. It has a catchment population of approximately forty million and is a referral point for the most severe pediatric patients. Overall, inpatient pertussis patients peaked in the hot season, often from May to July (Fig. 1). Pertussis patients during this season accounted for nearly half of all patients occurring year-round and were more than 1.5 times as many as those in the mild season (Table 2). The multivariate linear model indicated a 21-percent increase in pertussis patients (Table 3).

Region-specific analyses of patients also provided comparable results. The central, more urbanized areas, where Hanoi is the capital of Vietnam, followed the same trends as the rural areas, as presented in Table 2. These trends of pertussis are similar to those in Australia and 30 European countries, despite climatic differences [16, 17]. Regarding the incidence of pertussis patients per 1,000 inpatients in VNCH, the peaks varied by year: February (mid-season) in 2015 and 2017 or May (hot season) in 2016 and 2018. Data from the USA have shown similar trends [2], demonstrating that seasonal patterns varied across age groups in North Vietnam, although infants constituted 88% of the studied cases.

The prevalence of pertussis is higher in the hot season, which may be linked to the increased use of air conditioning systems [18–20]. Moreover, the trend of the pertussis incidence (the number of cases per 1,000 VNCH inpatients) in Fig. 3 resembled that of hospitalization in Fig. 1; both directions showed peaks in the hot season (May to July). The multivariable linear regression of the pertussis incidence by season also indicated comparable results regarding the vaccination rate and the average age (Table 3). It shows that pertussis hospitalization did increase during the hot season, even when controlling for other factors, including the vaccination rate. Studies conducted in other countries have also demonstrated that the majority of reported pertussis cases occurred during the summer season when temperatures were high [2, 21–24]. Further studies should investigate the association between household habits and pertussis incidence to provide more solid evidence for potential public health interventions.

We found that the proportion of severe pertussis, the need for mechanical ventilation, and the mortality rate due to pertussis in the mild season were significantly higher than those in the hot season (Table 2). Temperature and precipitation are the two factors that make the most significant differences between mild, hot, and warm seasons. According to the WHO, the mortality rate caused by respiratory infections in many countries during winter is 10–25% higher than that during summer [2]. Our current study also showed that the incidence of pertussis and the rate of severe cases in mild and warm seasons with cooler weather were higher than in the hot season; under 2-month-old children in the mild season accounted for the highest rates (Table 2). Other studies also demonstrated an increase in severe cases among younger children [25]. They considered household contact during the Tet holiday to be a factor that increases the risk of getting pertussis among young children, especially those younger than the age of vaccination. This suggests that vaccination rates should be increased among adults, especially women preparing for pregnancy or childbearing. VNCH and other hospitals in Vietnam have been facing shortages of devices to monitor the air temperature and humidity from ventilators. Thus, there should be greater attention to moisture control, thermal comfort, and natural ventilation in patients’ rooms, both at home and in the hospital, especially in the intensive care unit. These precautions should be emphasized explicitly in the mild season to improve the quality of care and treatment [18–20, 26].

A seasonal cycle was more evident in 2015 and 2017 (Fig. 2). Despite the high vaccination coverage in North Vietnam (usually more than 90%), outbreaks occur biennially, similar to those in countries such as Australia, the Netherlands, and Nigeria [10, 16, 27]. Moreover, nearly 64% of cases were under three months old, of whom under 2-month-old infants, who are ineligible for vaccination, constituted approximately 70%. The possible reasons behind this may include the delay of the first vaccine dose and the lack of immunization before pregnancy or among women of childbearing age [13, 28]. Since 2015, the VNCH has started implementing measures to encourage doctors to increase the frequency of diagnostic tests for pertussis and other infectious diseases. At the end of 2016, the hospital conducted an intervention to improve the early detection of pertussis. Some studies have indicated that neonates’ clinical signs and symptoms are more typical; therefore, diagnoses in this group were better than those in the others [21, 29].

Additionally, the pertussis admissions at VNCH over the four years between 2014 and 2017 had the same age distribution: the highest number of cases (approximately 40%) were infants under two months old. This made us concerned about the success of the testing policy. Nevertheless, the delay in diagnosis from onset to the test date decreased significantly (*p* < 0.05) from 11 days before interventions (in 2015 and 2016) to 10 days after interventions (in 2017 and 2018). Contrary to what we expected, the promotion of early testing has not decreased the proportion of severe pertussis, the number of fatalities, or the duration of hospitalization among pertussis inpatients (Table 1). Possibly due to the fact that the diagnosis time is still lengthy, despite being shortened, and the diagnosis time for infectious diseases such as whooping cough remains too long at 10 days, the number of severe cases has still not decreased. Therefore, parents must be equipped with better knowledge of the signs and symptoms of pertussis so that they can distinguish pertussis infection from other respiratory diseases and have their children diagnosed early at the hospital.

The Red River Delta region had the highest number of pertussis cases in our study. It is one of Vietnam’s most important economic centers, where the capital city of Ha Noi is located and where higher education and modern health services are most easily accessible. The North Delta region had the highest number of pertussis cases, accounting for 70% of all cases nationwide (Table 2). Apart from its high population density, another possible explanation for this higher incidence is that it has the main transport route network in North Vietnam. These factors may make the risk for pertussis transmission in this region higher than in other places. In addition, our study results showed two more reasons for higher transmission in the Red River Delta region: the Tet holiday gatherings and the use of air conditioning systems in the hot season. Therefore, health education and communication interventions should be conducted in Vietnamese settings, especially in the Red River Delta Region, to provide parents or child caregivers with better knowledge to increase the use of natural ventilation and change behaviors that may harm children, for example, kissing infants or feeding them foods that are pre-masticated.

### Limitation

Many studies on national disease surveillance have two gaps in their data: many cases are not diagnosed because of mild signs and symptoms, and stakeholders’ interests in the health system are not mentioned [30]. Pertussis is endemic in Vietnam under the “National Notifiable Disease Surveillance System (NNDSS),” governed by Circular 54 of the Ministry of Health. Hospitals in Vietnam report most data on pertussis cases in the system. This study used hospital-based data collected from patients admitted to VNCH as a tertiary hospital in North Vietnam, constituting 70–90% of all patients in North Vietnam for the same period (data from NSS [12]). However, severe and critical cases among hospitalizations accounted for a sizable proportion, and most patients admitted to VNCH came from northern provinces and cities. This proportion, therefore, might be higher than that of the general population. This may compromise the generalization of the study results. Further studies should be conducted to provide additional evidence for appropriate interventions.

## Conclusion

The transmission of this disease reached a peak during the hot season. However, the severity and mortality increased in the mild season. These findings are expected to provide further evidence for developing appropriate interventions to reduce the severity and mortality of pertussis, especially in the mild season.

This study utilized hospital-based data of patients exclusively who were admitted to the VNCH. Further research efforts may benefit from including multiple hospitals to expand the sample size of patients with pertussis. Additionally, such surveillance data need to be regularly analyzed to monitoring and projecting epidemic patterns, and estimate pertussis rates among the general population by seasons or different time points.

## Data Availability

The datasets used and/or analyzed during the current study are available from the corresponding author on reasonable request.

## Abbreviations

ECMO: Extracorporeal Membrane Oxygenation
IQR: Interquartile Range
MOH: Ministry of Health
VNCH: Vietnam National Children’s Hospital
WHO: World Health Organization

## References

[1] Pinkbook Course Book: Epidemiology of Vaccine Preventable Diseases | CDC. 2021. https://www.cdc.gov/vaccines/pubs/pinkbook/index.html. Accessed 6 Dec 2022.

[2] Pertussis (Whooping Cough). Outbreaks | CDC. 2023. https://www.cdc.gov/pertussis/outbreaks.html. Accessed 30 Nov 2023.

[3] Pertussis - Annual Epidemiological Report for 2016. 2018. https://www.ecdc.europa.eu/en/publications-data/pertussis-annual-epidemiological-report-2016. Accessed 30 Nov 2023.

[4] Ministry of Health. Vietnam Health Statistics Yearbook., 2017. Ha Noi: Medical Publishing House; 2018.

[5] El Guerche-Séblain C, Caini S, Paget J, Vanhems P, Schellevis F (2019). Epidemiology and timing of seasonal influenza epidemics in the Asia-Pacific region, 2010–2017: implications for influenza vaccination programs. BMC Public Health.

[6] Factsheet about seasonal influenza. 2017. https://www.ecdc.europa.eu/en/seasonal-influenza/facts/factsheet. Accessed 30 Nov 2023.

[7] Bakker KM, Martinez-Bakker ME, Helm B, Stevenson TJ (2016). Digital epidemiology reveals global childhood disease seasonality and the effects of immunization. Proc Natl Acad Sci U S A.

[8] Steele L, Orefuwa E, Dickmann P (2016). Drivers of earlier infectious disease outbreak detection: a systematic literature review. Int J Infect Dis.

[9] Conducting a Field Investigation | Epidemic Intelligence Service | CDC. 2023. https://www.cdc.gov/eis/field-epi-manual/chapters/Field-Investigation.html. Accessed 30 Nov 2023.

[10] Seasonal patterns in time series of pertussis - PubMed. https://pubmed.ncbi.nlm.nih.gov/19327200/. Accessed 30 Nov 2023.

[11] Martinez ME (2018). The calendar of epidemics: seasonal cycles of infectious diseases. PLoS Pathog.

[12] Ministry of Health. Vietnam Health Statistics Yearbook., 2018. Ha Noi: Medical Publishing House; 2019.

[13] Pham NTH, Le NDT, Le NK, Nguyen KD, Larsson M, Olson L (2020). Pertussis epidemiology and effect of vaccination among diagnosed children at Vietnam, 2015–2018. Acta Paediatr.

[14] Yerdessov S, Abbay A, Makhammajanov Z, Zhuzzhasarova A, Gusmanov A, Sakko Y (2023). Epidemiological characteristics and seasonal variation of measles, pertussis, and influenza in Kazakhstan between 2010–2020 years. ELECTRON J GEN MED.

[15] That’s how warm it is in Vietnam: 28.9°C on average per year and over 1750 hours of sunshine! Worlddata.info. https://www.worlddata.info/asia/vietnam/climate.php. Accessed 2 Dec 2023.

[16] Leong RNF, Wood JG, Turner RM, Newall AT (2019). Estimating seasonal variation in Australian pertussis notifications from 1991 to 2016: evidence of spring to summer peaks. Epidemiol Infect.

[17] Pertussis - Annual Epidemiological Report for 2018. 2020. https://www.ecdc.europa.eu/en/publications-data/pertussis-annual-epidemiological-report-2018. Accessed 30 Nov 2023.

[18] Airborne Diseases: Types, Prevention, and More. https://www.healthline.com/health/airborne-diseases. Accessed 30 Nov 2023.

[19] Randazzo T, De Cian E, Mistry MN (2020). Air conditioning and electricity expenditure: the role of climate in temperate countries. Econ Model.

[20] Correia G, Rodrigues L, Gameiro da Silva M, Gonçalves T (2020). Airborne route and bad use of ventilation systems as non-negligible factors in SARS-CoV-2 transmission. Med Hypotheses.

[21] Ghorbani GR, Zahraei SM, Moosazadeh M, Afshari M, Doosti F (2016). Comparing Seasonal Pattern of Laboratory confirmed cases of Pertussis with clinically suspected cases. Osong Public Health Res Perspect.

[22] Kwon HJ, Yum SK, Choi UY, Lee SY, Kim JH, Kang JH (2012). Infant Pertussis and Household Transmission in Korea. J Korean Med Sci.

[23] Wendelboe AM, Njamkepo E, Bourillon A, Floret DD, Gaudelus J, Gerber M (2007). Transmission of Bordetella pertussis to young infants. Pediatr Infect Dis J.

[24] de Greeff SC, Mooi FR, Westerhof A, Verbakel JMM, Peeters MF, Heuvelman CJ (2010). Pertussis disease burden in the household: how to protect young infants. Clin Infect Dis.

[25] Shi T, Wang L, Du S, Fan H, Yu M, Ding T (2021). Mortality risk factors among hospitalized children with severe pertussis. BMC Infect Dis.

[26] Climate change and human health - risks and responses. https://www.who.int/publications-detail-redirect/924156248x. Accessed 30 Nov 2023.

[27] Abubakar A, Dalhat M, Mohammed A, Ilesanmi OS, Anebonam U, Barau N et al. Outbreak of suspected pertussis in Kaltungo, Gombe State, Northern Nigeria, 2015: the role of sub-optimum routine immunization coverage. Pan Afr Med J. 2019;32 Suppl 1:9. 10.11604/pamj.supp.2019.32.1.13352 PMC6441470 30949284

[28] Pinkbook. Pertussis | CDC. 2022. https://www.cdc.gov/vaccines/pubs/pinkbook/pert.html. Accessed 30 Nov 2023.

[29] Dien MT. Characteristics of pertussis patient in Vietnam national childrens hospital in 2015. Vietnam J Prev Med. 27:69–76.

[30] O’Brien JA, Caro JJ (2005). Hospitalization for pertussis: profiles and case costs by age. BMC Infect Dis.

